# Antiangiogenic properties of Koetjapic acid, a natural triterpene isolated from *Sandoricum koetjaoe *Merr

**DOI:** 10.1186/1475-2867-11-12

**Published:** 2011-04-27

**Authors:** Zeyad D Nassar, Abdalrahim FA Aisha, Mohamed BK Ahamed, Zhari Ismail, Khalid M Abu-Salah, Salman A Alrokayan, Amin Malik Shah Abdul Majid

**Affiliations:** 1 Department of Pharmacology, School of Pharmaceutical Sciences, Universiti Sains Malaysia, Minden 11800, Pulau Penang, Malaysia; 2 Department of Pharmaceutical Chemistry, School of Pharmaceutical Sciences, Universiti Sains Malaysia, Minden 11800, Pulau Penang, Malaysia; 3 The chair of Cancer Targeting and Treatment, Biochemistry Department and King Abdullah Institute for Nanotechnology, King Saud University, 2454, Riyadh 11451, Saudi Arabia; 4 Australian Institute for Bioengineering and Nanotechnology, University of Queensland, Brisbane, Qld 4072, Australia

## Abstract

**Background:**

Angiogenesis, the formation of new blood vessels, has become an important target in cancer therapy. Angiogenesis plays an important role in tumor growth and metastasis. Koetjapic acid (KA) is a seco-A-ring oleanene triterpene isolated from *S. koetjape*. The solvent extract of this plant species was shown previously to have strong antiangiogenic activity; however the active ingredient(s) that conferred the biological activity and the mode of action was not established. Given the high concentration of KA in *S. koetjape*, an attempt has been made in this study to investigate the antiangiogenic properties of KA.

**Results:**

Treatment with 10-50 μg/ml KA resulted in dose dependent inhibition of new blood vessels growth in *ex vivo *rat aortic ring assay. KA was found to be non-cytotoxic against HUVECs with IC_50 _40.97 ± 0.37 μg/ml. KA inhibited major angiogenesis process steps, endothelial cell migration and differentiation as well as VEGF expression.

**Conclusions:**

The non-cytotoxic compound, KA, may be a potent antiangiogenic agent; its activity may be attributed to inhibition of endothelial cells migration and differentiation as well VEGF suppression.

## Background

Angiogenesis research is the cutting edge technology that is currently being heavily exploited in the cancer field [1]. Angiogenesis research will probably change the face of medicine in the next decades; more than 500 million people worldwide are expected to benefit from pro- or antiangiogenesis treatments [2]. Angiogenesis is a process of new blood vessel development orchestrated by a range of angiogenic factors and inhibitors. This process is tightly regulated and self limiting in some cases such as wound healing, normal growth process and reproductive function [3]. In contrast, when this process is deregulated, diseases such as cancer, rheumatoid arthritis, obesity and diabetic blindness can be formed [2,4]. Angiogenesis plays an important role in cancer growth without which, tumors will be unable to expand beyond 1 to 2 mm^3 ^[5]. Cancer cells within the tumor will then use the newly formed blood vessels as a port to metastasize to other localities [6].

Since the interdependency and a close relationship between angiogenesis, cancer growth and metastasis has been well-established, much effort have been invested into development or discovery of antiangiogenic compounds to target cancer and variety of other angiogenic related ailments.

Angiogenesis inhibitors exhibited their activity via direct or indirect machineries. The direct angiogenesis inhibitors interfere with the endothelial cell functions such as proliferation, migration and differentiation. On the contrary, indirect angiogenesis inhibitors prohibit the pro-angiogenic communication between the tumor-cell and endothelial-cell compartments by decreasing of angiogenic signals expression, or interference with binding of these signals with the receptors on the endothelial cells.

**Figure 1:**
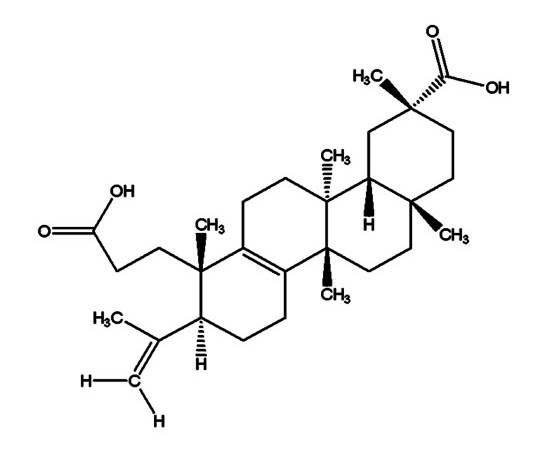
**Chemical structure of KA**. The structure of KA has been resolved by X-ray crystallography as described previously [18].

*S. koetjape *is a terpenoids-rich traditional medicinal plant belonging to the family Meliaceae, native to Malaysia, Cambodia and Southern Laos [7]. Our previous studies reported strong anti-angiogenic effect of n-hexane extract of *S. koetjape *on human colon cancer cell lines, however, the underlying mechanism was not known [8]. Koetjapic acid (KA) is a seco-A-ring oleanene triterpene isolated from *S. koetjape *(Figure 1). KA was found to have poor cytotoxicity on a number of human cancer cell lines and murine lymphocytic leukemia [7]. KA was also shown to have ichthyotoxic and chemopreventive effects [9]. In this study, an attempt has been made to investigate the antiangiogenic activity of KA *in vitro *and *in vivo*.

## Results

### KA inhibits the sprouting of microvessels in rat aortic rings

**Figure 2:**
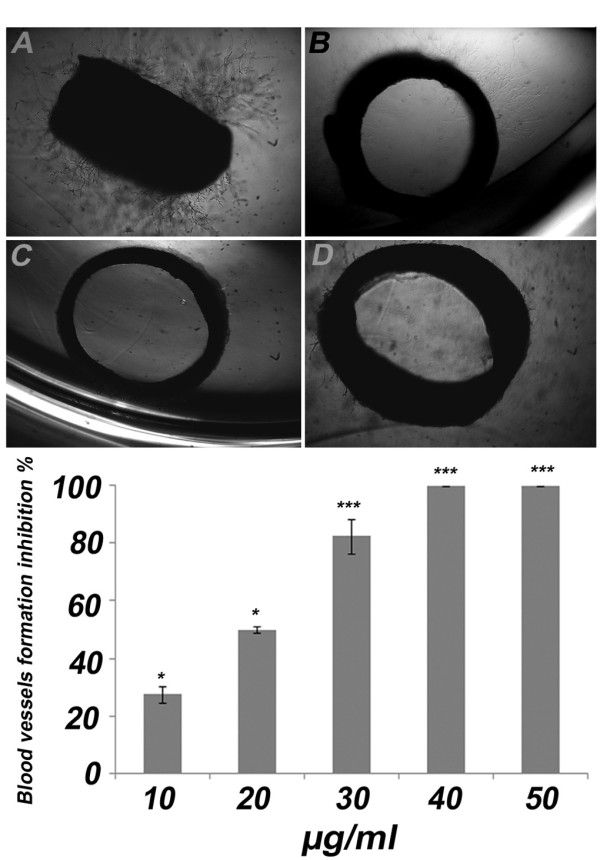
**Effects of KA on angiogenesis in rat aortic ring assay**. Effect of KA on microvessels formation in rat aortic rings. Explants treated with (A) 1% ethanol (B) 10 μg/ml (C) 40 μg/ml and (D) 100 μg/ml suramin as a positive control (4X). (E) The Dose response relationship of KA on rat aorta assay. Data was represented as mean ± SD (n = 3). *P <0.05 and ***P <0.001.

The anti-angiogenic potential of KA was investigated firstly in the rat aortic model. Figure (2A) shows the microvessels outgrowth from the untreated aortic rings. On the contrary, aortic rings treated with KA exhibited reduced outgrowth with IC_50 _16.8 ± 1.7 μg/ml (Figure 2B and 2C). The anti-angiogenic effect on explants of rat aorta showed a significant dose dependent relationship (*P *< 0.05). At 20 μg/ml, KA inhibited vascularisation by 50% and doubling the dose to 40 μg/ml led to a complete inhibition of angiogenesis by 100%. Suramin, which was used as a positive control showed almost 100% inhibition of microvessels outgrowth at 100 μg/ml (Figure 2D).

### Effect of KA on HUVECs Proliferation

**Figure 3:**
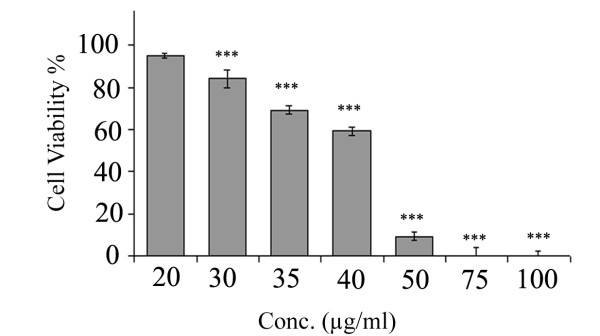
**Effects of KA on HUVECs proliferation**. Effects of KA on HUVECs viability. KA inhibits proliferation of HUVECs in dose dependent manner. Values are means of three experiments (n = 3). Data presented in mean ± SD.

KA was found non cytotoxic agent against HUVECs as the IC_50 _value was found to be 40.97 ± 0.37 μg/ml. At concentration of 20 μg/ml, KA causes no significant inhibition on HUVECs proliferation (*P *> 0.05). Figure (3) demonstrates the dose dependent activity of KA on HUVECs proliferation.

### KA Inhibits HUVECs Migration

**Figure 4:**
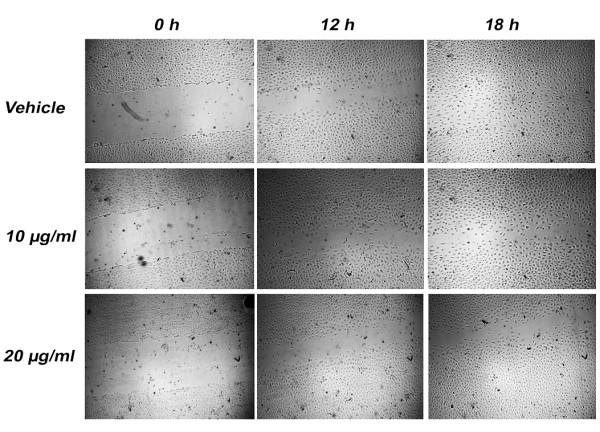
**Effects of KA on HUVECs Migration**. A scratch is created and then the cells were treated with 1% ethanol or 10 and 20 μg/ml of KA. At 0, 12 and 18 h, pictures of the wounds were captured at 4X magnification.

Effect of KA on endothelial cell migration was studied using endothelial cell migration wound healing assay. Significant reduction in HUVECs motility was achieved at 10 μg/ml with 6.9 ± 1.19% and 10.6 ± 2.31% inhibition at 12 and 18 h treatment, respectively (*P *< 0.05) (Figure 4). At 20 μg/ml, KA inhibited HUVECs migration by 27.3 ± 3.1% and 23.6 ± 1.28% after 12 h and 18 h incubation period, respectively (*P *< 0.001).

### KA Inhibits Differentiation of HUVECs on Matrigel Matrix

**Figure 5:**
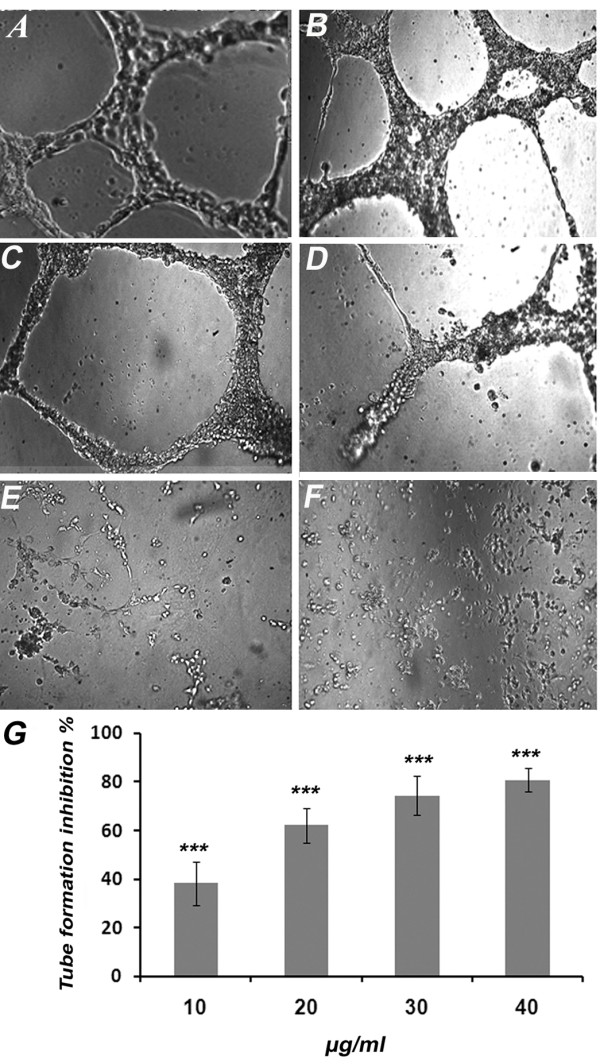
**Effects of KA on HUVECs Differentiation**. Effect of KA on matrigel tube formation of endothelial cells. Cells treated with (A) 1% ethanol (B) 10 μg/ml (C) 20 μg/ml and (D) 30 μg/ml (E) 40 μg/ml and (F) 100 μg/ml suramin as a positive control (4X). (G) The Dose response relationship of KA on rat aorta assay. The inhibitory effect was found to be in dose response manner. Data was represented as mean ± SD (n = 3). ***P <0.001.

In normal setting, endothelial cells will form tube-like structure networks in a matrigel matrix within 6 h. Treatment of HUVECs with KA inhibited the growth factors induced differentiation in a dose-dependent manner. At lower concentrations, KA reduced the area occupied by the network structures and incomplete and broken tubules were formed (Figure 5). At 40 μg/ml, KA completely abrogated endothelial tube formation. The IC_50 _value was 14.07 ± 2.74 μg/ml comparable with that of the suramin positive control.

### KA Inhibits VEGF Expression

KA caused significant inhibition of VEGF level in HUVECs. KA at 20 μg/ml suppressed VEGF expression by 30.68%. The VEGF concentration in cell lysates of treated HUVECs at 20 μg/ml was 262 ± 22.37 pg/ml and was significantly lower than untreated cells which was 378 ± 13.09 pg/ml (*P < 0.001)*.

### *In vivo *CAM Assay

**Figure 6:**
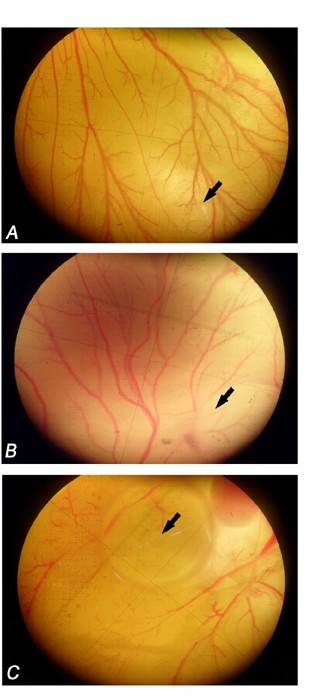
**Effects of KA on neovascularization in CAM assay**. Effect of KA on neovascularization in chorioallantoic membrane of chick embryo. The embryos were treated for 24 h with (A) 1% ethanol as a negative control, (B) 50 μg/ml and (C) 100 μg/ml. The pictures were captured under dissecting microscope. Arrows indicate the place of agarose discs application. KA inhibited the neovascularisation in a dose-dependent manner by 41.8 ± 7.2% and 64.9 ± 8.7% at 50 and 100 μg respectively. Data presented in mean ± SD.

Vascularisation in chick embryo was significantly inhibited by KA at 100 and 50 μg/disc. The displayed images in Figure 6 show normal vasculature pattern in the untreated CAMs with primary, secondary and tertiary vessels and dendritic branching pattern. On the other hand, the 100 and 50 μg KA-treated CAM showed distorted architecture in the vasculature. The number of the blood vessels was decreased significantly in treated CAMs. KA inhibited the formation of new blood vessels significantly by 41.8 ± 7.2% and 64.9 ± 8.7% at 50 and 100 μg respectively, when compared to the control.

## Discussion

Recently more efforts have been concentrated on synthesis or discovery of non-cytotoxic compounds that have antiangiogenic and anti-neoplastic activity. This treatment approach decreases the side effects that accompany the classical chemotherapeutics drugs. Angiogenesis involves a series of processes including activation of the endothelial cells, degradation of the basement membrane, endothelial cell migration, proliferation of the endothelial cells, vessels elongation, vessels branching, vasodilatation, formation of basement membrane, acquisition of pericyte, and re-modelling [10]. Cancer colonies remain undetected and dormant for several years, and under certain circumstances the tumor cells switch into angiogenic phenotype, induce formation of new blood vessels, then start to grow and metastasize to other localities [11]. The angiogenic switch takes place when the positive angiogenic factors such as VEGF outweigh the negative factors such as endostatin [12]. Therefore, the angiogenic switch may result from over production of VEGF. Many studies have been focused on screening novel non-cytotoxic compounds or extracts from natural products but have antiangiogenic properties [13,14]. KA has found to have antibacterial, anti-inflammatory, anti-tumor promoting activity and DNA polymerase inhibition properties [9,15-17]. Till now, there are no reports about antiangiogenic activity of KA. Previously we have shown that the hexane extract of *Sandoricum koetjape *have a strong antiangiogenic activity *ex vivo *[8]. Fractionation and crystallization of the extract has led to the isolation of KA [18].

In this study, a battery of well established angiogenesis assays was employed to evaluate the antiangiogenic activity of KA. The results showed that KA perturbed the new blood vessels formation in rat aortic ring explants with IC_50 _of 16.8 ± 1.7 μg/ml (Figure 2). The effect of KA on HUVECs proliferation has been conducted to measure if the antiangiogenic activity observed was due to cytotoxic activity towards the endothelial cells or anti-proliferative activity. The MTT results showed that KA is not cytotoxic towards the endothelial cells and the IC_50 _value was at 40.97 ± 0.37 μg/ml. Furthermore, it has no significant effect on cells proliferation at 20 μg/ml (*P *> 0.05) (Figure 3). The cytotoxicity results are compatible with the previous work on anticancer activity of this compound [7]. On the other hand, KA inhibited 100% of the angiogenesis process in the *ex vivo *rat aortic ring model at a dose of 40 μg/ml and around 50% at a dose of 20 μg/ml of KA. Taken together, the results of rat aortic ring assay and the MTT assay on HUVECs confirmed that the antiangiogenic effect demonstrated by KA is not due to the cytotoxic nature of the compound, but may be more related to inhibition of one or more of other angiogenesis cascade.

In order to investigate the angiogenesis inhibitory mechanisms of KA, we have studied its effects on three major steps in angiogenesis namely, endothelial cells migration, endothelial cells differentiation and VEGF expression. KA inhibits all of these processes significantly at a dose 20 μg/ml at a dose that is below the cytotoxic concentration. At 20 μg/ml, the compound inhibits 31% of VEGF expression in HUVECs. VEGF is regarded as the major growth factor that triggers the angiogenesis event. It is responsible for triggering various steps in the angiogenesis cascade such as proliferation, migration and cell survival [19]. VEGF has been found significantly up-regulated at the levels of RNA and protein in most types of cancer. The high concentration of VEGF in cancer patients is associated with poor prognosis as well as with low survival [20]. During the angiogenesis cascade, the extracellular matrix and vascular basement membrane are degraded, permitting the endothelial cells to migrate into the perivascular space in the direction of angiogenic stimuli. At a concentration of 20 μg/ml, KA inhibited HUVECs migration by 27.3 ± 3.1% and 23.6 ± 1.28% after 12 h and 18 h incubation period, respectively (*P <*0.001) (Figure 4). Nitric oxide (NO) has been identified as an endothelium-derived relaxing factor (EDRF) [21], which induces endothelial cell proliferation and migration [22,23]. Thus, no wonder that EDRF/NO has been revealed to play a crucial role in angiogenesis process regulation [24]. VEGF has been shown to stimulate endothelial NO production [25-27], additionally, many reports have shown that eNOS lies downstream of VEGF and both are under the target of HIF-1 transcriptional factor [28]. The ability of KA to inhibit the endothelial cells migration may be implicated to inhibition of VEGF production and consequently inhibition of nitric oxide signaling.

After cells migrate into the perivascular space, HUEVCs differentiate so their shapes change in a way that facilitates the adherence amongst the cells to form a lumen (tube-like structure) [29]. KA inhibited the formation of such tube like structures in a dose dependent relationship (Figure 5). At the concentration equal to IC_50 _against HUVECs proliferation (40 μg/ml), KA inhibited tube formation by 91%, and at the non-toxic dose (20 μg/ml), tube formation inhibition was around 60%. Endothelial cells differentiation requires activation of VEGFR-1 (FLt-1) [30]. The significant inhibition of VEGF expression by KA may play role in VEGFR-1 activation and consequently inhibition of HUVECs differentiation.

The final step in this study was to test if the KA can exhibit its antiangiogenic properties *in vivo *as it did in *in vitro *and *ex vivo *assays. The vascularisation in chick embryo was significantly inhibited by the compound at 50 μg and 100 μg (Figure 6). The results of this study show that KA inhibited many crucial steps of the angiogenesis process hence it may be useful in treating angiogenesis related ailments such as cancer.

## Conclusions

In conclusion, this study demonstrates that KA can inhibit neovascularisation by affecting VEGF expression, endothelial cell migration and endothelial tube formation.

## Material and methods

### Cell lines and Culture Conditions

Human umbilical vein endothelial cells (HUVECs); Catalogue number (8000) was purchased from ScienCell, USA. HUVECs were maintained in ECM medium supplemented with 5% HIFBS, 1% PS and 1% ECGS. Cells were cultured in a 5% CO_2 _in a humidified atmosphere at 37°C.

### Chemicals and Reagents

Endothelial Cell Medium (ECM) supplied with endothelial cell growth supplements (ECGS) was purchased from ScienCell, USA. trypsin and heat inactivated foetal bovine serum (HIFBS) were obtained from GIBCO, UK. Human VEGF assay kit was obtained from Raybio, USA. Phosphate buffered saline (PBS), penicillin/streptomycin (PS) solution, MTT reagent, suramin, amphotericin B, aprotinin, 6-aminocaproic acid, L-glutamine, thrombin, gentamicin were purchased from Sigma-Aldrich, USA. Fibrinogen was supplied by Calbiochem, USA. Matrigel matrix (10 mg/ml) was purchased from BD Bioscience, USA. Other chemicals and solvents were of analytical grade.

### Experimental Animal

The 12-14 weeks old Sprague Dawley male rats were obtained from the animal house facility of USM. All procedures were carried out in accordance with the guidelines of USM Animal Ethical Committee. The animals were kept for one week in transient animal house (School of Pharmaceutical Sciences, USM) prior to the experiment. The animals were kept in well ventilated cages at 12 h light cycle with food and water ad libitum. The animals were humanely sacrificed under CO_2_.

### Koetjapic Acid

KA was isolated the from *S. koetjape *stem bark as described previously [18]. Briefly, 10 g of n-hexane extract was crystallized at -20°C in 50 ml methanol: acetone at 1:1 v/v. The collected crystals (500 mg) were re-crystallized in chloroform by solvent evaporation to give 400 mg colourless prism-shaped crystals (0.2%). The three dimensional structure of the compound was elucidated by X-ray crystallography as described previously [18].

KA was dissolved in ethanol to obtain 10 mg/ml stock solution and stored at 4°C. For drug treatment, KA was diluted in indicated culture medium at the indicated concentrations in each experiment.

### Cell Proliferation Assay

Cytotoxicity of the KA was evaluated by MTT assay. Cells were treated for 48 h with KA or 1% ethanol as a negative control. Viability of cells were determined by MTT test as described previously [31]. Assay plates were read using a microtiter plate reader (Hitachi U-2000, Japan) at *A*_*570*_. The results are presented as percent viability to the negative control (n = 3).

### *Ex vivo *Rat Aortic Ring Assay

The rat aortic ring assay was carried out according to techniques established by Brown et al (1996) with slight modification [32]. In brief, aortic rings (1 mm thickness) taken from thoracic aortas of 12-14 weeks old male Sprague Dawley rats were seeded individually in 48-wells plate in 300 μl serum free M199 media containing 3 mg ml^-1 ^fibrinogen and 5 mg ml^-1 ^aprotinin. 50 NIH U ml^-1 ^thrombin in 0.15 M NaCl were added in each well. After 90 min incubation at 37°C, various concentrations of KA dissolved in 0.3 ml M 199 medium supplemented with 20% HIFBS, 0.1% έ-aminocaproic acid, 1% L-Glutamine, 2.5 μg/ml amphotericin B, and 60 μg/ml gentamicin, were added to each well. Suramin and 1% ethanol were used as positive and negative controls, respectively. On day four, the medium was replaced with a fresh one containing the compound. On day five, aortic rings were photographed at 4x magnification using an inverted light microscope (Olympus). The angiogenic response was determined by measuring the distance of blood vessels outgrowth from the primary tissue ex-plants using the same instrument with the aid of Leica Quin software package [33]. The results are presented as mean percent inhibition to the negative control ± SD, (n = 3).

### Migration Assay

The assay was carried out as prescribed previously [34]. Briefly, HUVECs were plated in 6 well plates till the formation of a confluent monolayer after which a wound was created with 200 μl micropipette tips. The detached cells were removed by washing twice with PBS and the plates were treated with KA. The wounds were photographed after 12 and 18 h, and the width of the cell-free wounds was measured using an inverted light microscope supplied with Leica Quin computerized imaging system. 10 fields per well were photographed and minimum of 30 readings per field were taken. The results are presented as mean percentage of migration inhibition compared to control ± SD, (n = 3).

### Tube Formation Assay

The ability of HUVECs to form tube-like structures was investigated on a matrigel matrix. In Brief, the matrigel matrix was allowed to polymerize for 45 min at 37°C and 5% CO_2_. HUVECs were trypsinized and seeded (3 × 10^4 ^cells per well) in 100 μl of ECM containing various concentrations of KA in triplicates. After 6 h tubular structures were imaged under an inverted light microscope at 4X magnification. The quantitative assessment of tube formation inhibition was achieved by measuring the area occupied by tubular structures using the Scion Image analysis program [35]. The results are presented as a mean percentage of inhibition ± SD, (n = 3).

### Determination of VEGF Concentration in HUVECs Lysates

VEGF concentration was determined using a human VEGF-165 ELISA kit (Raybio, USA) as per the manufacturer's instructions. According to the manufacturer, the minimum detectable concentration of VEGF is less than 20 pg/ml. Briefly, 60-70% confluent cultures of HUEVCs were treated with KA at 20 μg/ml for 6 h, and cell lysates were prepared using cell lysis buffer provided along with the kit. At the time of the experiment a calibration curve of VEGF standard was prepared. The concentration of VEGF in cell lysates was calculated using the log-log regression equation of the best fit line of the standard calibration curve; (y = 0.0099 × ^0.6137^, *R*^2 ^= 0.996). The experiment was repeated twice in triplicates.

### *In vivo *CAM Assay

Antiangiogenic effect of KA was investigated *in vivo *in CAM assay as mentioned previously [36]. Five Day-old fertilized eggs were obtained from local hatchery. 5 ml of albumin was aspirated and the eggs were incubated horizontally to allow the CAM to detach from the shell. KA was prepared in agarose 1.2% discs at 100 or 50 μg/disc. Discs containing the vehicle only (ethanol) were used as negative control. Then a small window opening was made in the shell, and the discs were directly applied onto the CAM. The square opening was covered with sterilised surgical tape and the embryos were incubated for 24 h. The CAMs were photographed under a dissecting microscope and blood vessels in each CAM were counted. The results are presented as a mean percentage of inhibition comparing to control ± SD, (n = 3).

### Statistical Analysis

Results were presented as means ± SD and differences between groups were compared by the one way ANOVA and considered significant at *P *< 0.05, 0.01 or 0.001. The statistical analysis was carried out by using SSPS edition 16.0.

## Competing interests

The authors declare that they have no competing interests.

## Authors' contributions

ZDN: Wrote part of the paper, conducting the KA purification, conducting the cell culture work and rat aortic ring assay. AFAA: Conducting the tube formation assay, design the experiment. MBKA: Conducting the in vivo cam assay. KMA: Participated in research design.SAA: Analyzed the data. AMSA: Participated in research design, wrote part of the paper. All authors read and approved the final manuscript.
